# A Mendelian Randomization Study of Plasma Homocysteine and Multiple Myeloma

**DOI:** 10.1038/srep25204

**Published:** 2016-04-29

**Authors:** Yang Xuan, Xiao-Hong Li, Zhong-Qian Hu, Zhi-Mei Teng, Dao-Jun Hu

**Affiliations:** 1 Key Laboratory of Environmental Medicine Engineering, Ministry of Education, Department of Epidemiology & Biostatistics, School of Public Health, Southeast University, Nanjing 210009, China; 2 Department of Rehabilitation Medicine, First Affiliated Hospital, Chongqing Medical University, Chongqing 400016, China; 3 Department of Ultrasound, Zhongda Hospital, Southeast University, Nanjing 210009, China; 4 Department of Clinical Laboratory, Xin Hua Hospital Affiliated to Shanghai Jiao Tong University School of Medicine, Chongming Branch, Shanghai 202150, China

## Abstract

Observational studies have demonstrated an association between elevated homocysteine (Hcy) level and risk of multiple myeloma (MM). However, it remains unclear whether this relationship is causal. We conducted a Mendelian randomization (MR) study to evaluate whether genetically increased Hcy level influences the risk of MM. We used the methylenetetrahydrofolate reductase (*MTHFR*) C677T polymorphism as an instrumental variable, which affects the plasma Hcy levels. Estimate of its effect on plasma Hcy level was based on a recent genome-wide meta-analysis of 44,147 individuals, while estimate of its effect on MM risk was obtained through meta-analysis of case-control studies with 2,092 cases and 4,954 controls. By combining these two estimates, we found that per one standard-deviation (SD) increase in natural log-transformed plasma Hcy levels conferred a 2.67-fold increase in risk for MM (95% confidence interval (CI): 1.12–6.38; *P* = 2.7 × 10^−2^). Our study suggests that elevated Hcy levels are causally associated with an increased risk of developing MM. Whether Hcy-lowering therapy can prevent MM merits further investigation in long-term randomized controlled trials (RCTs).

Multiple myeloma (MM) is a malignant disease characterized by proliferation of clonal plasma cells in the bone marrow and typically accompanied by the secretion of monoclonal immunoglobulins that are detectable in the plasma or urine, causing anemia, pathological fracture, and the clinical symptoms of renal failure. Among other plasma cell dyscrasias, such as Waldenström’s macroglobulinaemia and primary amyloidosis, multiple myeloma is the second most frequent haematological malignancy with an age-adjusted incidence of six per 100,000 per year in the USA and Europe^1^,^2^. However, the cause of the MM is still largely unknown. Risk factors such as age, positive family history, smoking, alcohol consumption, ionizing radiation, industrial occupation, and obesity have been reported for the development of MM^3^,^4^. Because treatments for MM are limited, the best approach to reduce mortality and morbidity is primary prevention through modification of acquired risk factors.

Homocysteine (Hcy), a sulfur-containing amino acid, is formed in the demethylation of the essential amino acid methionine^5^. Previous studies have demonstrated that high plasma Hcy concentration is an independent risk factor for cerebrovascular, peripheral vascular, and cardiovascular disease^6^,^7^. Furthermore, observational studies showed that plasma levels of Hcy as well as its metabolizing factors were associated with the overall risk of cancer^8^,^9^,^10^,^11^. However, Hcy level is also related to smoking status, blood pressure, and social class. Thus, this relation could be subject to residual confounding, reverse-causality bias, or both^11^. In view of these, we conducted a Mendelian randomization analysis to assess whether elevated Hcy level is causally associated within creased risk of MM.

In the absence of evidence from high-quality randomized controlled trials (RCTs), the principles of Mendelian randomization (MR) can be applied to strengthen or refute the causality of biomarkers in disease etiology. MR is a study design in which genetic variants are served as instrumental variables for estimating the unconfounded effect of an exposure (for example, Hcy) on a disease (for example, stroke)^11^. This approach, which is conceptually similar to a RCT, is based on the principle that genetic variants are randomly allocated at meiosis, and consequently these genetic variants are independent of many factors that bias observational studies, such as confounding and reverse causation. MR methods have been used previously to investigate the influence of type 2 diabetes(T2D) and fasting glucose (FG) on coronary heart disease(CHD) risk, which supporting a causal relationship between T2D and CHD^12^. MR methods may be of particular relevance for understanding the etiology of MM since the date of disease onset is often poorly recognized clinically and MR studies assess the effect of lifetime exposures.

MR analyses using methylenetetrahydrofolate reductase (*MTHFR*) C677T polymorphism as an instrument variable have been carried out in the past^11^,^13^,^14^,^15^.The researchers provided evidence from MR that plasma Hcy level is causally related to stroke, schizophrenia, T2D and offspring birth weight. In the present study, we provide evidence on the presence, direction, and magnitude of a causal effect of plasma Hcy levels on MM risk by performing a MR study based on the *MTHFR* C677T polymorphism as an instrumental variable.

Trial sequential analysis (TSA) is an approach that provides the required information size in meta-analyses. Under the premise of not expanding the type I error, we can earlier draw a conclusion using TSA, which can terminate defects of invalid test and save medical resources, compared to the traditional meta-analysis. And using TSA can make our result of meta-analysis be more stable and reliable^16^.

## Results

### Study selection and characteristics

**Figure 1:**
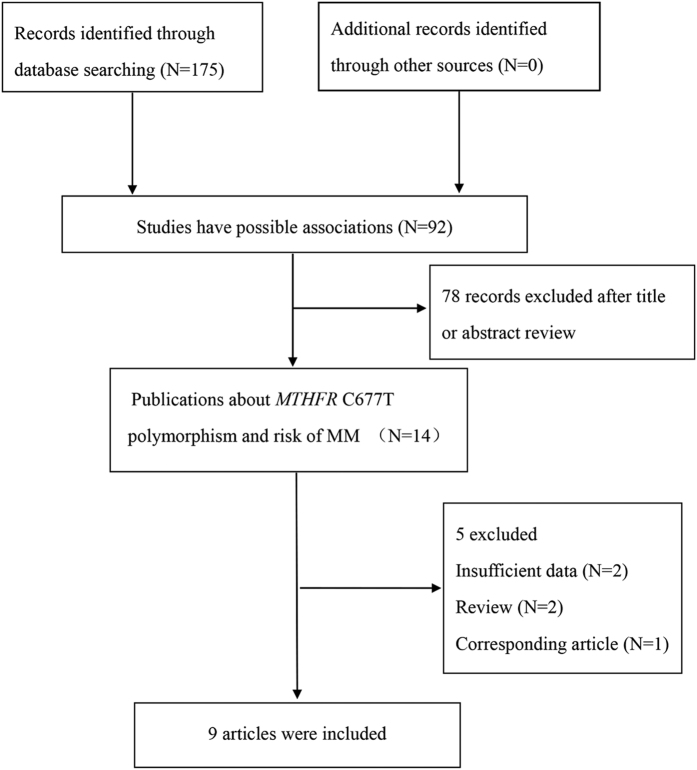
Flow diagram of study selection. The terms “N” in the boxes represent the number of corresponding studies.

The process of literature retrieval and exclusion was shown in Fig. 1. The literature search identified a total of 92 potential articles related to *MTHFR* C677T polymorphism and risk of MM. 78 articles were excluded after reading abstract and title. Then, 5 articles were excluded once again due to insufficient data, review or corresponding article. Finally, 9 studies with a total of 2,092 cases and 4,954 controls, were included in our meta-analysis^17^,^18^,^19^,^20^,^21^,^22^,^23^,^24^,^25^. The main characteristics of included studies were shown in Table 1. Among those 9 studies, 6 studies were carried out in Caucasians, while 3 were in Asian populations. Most of those included studies used RFLP-PCR to test the genotype of *MTHFR* C677T polymorphism, and a small part of which were genotyped by Microarray or TaqMan assay. Genotypes distribution in the controls of all included studies were in agreement with HWE (Hardy Weinberg Equilibrium).

### Association of *MTHFR* C677T polymorphism with risk of multiple myeloma

**Figure 2:**
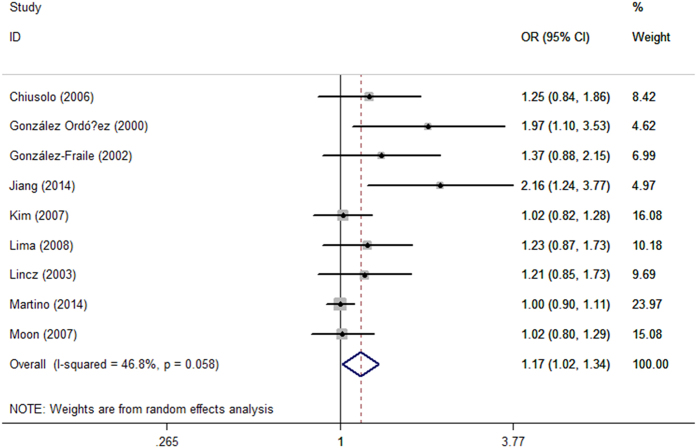
Forest plots of the risk of MM associated with the *MTHFR* C677T polymorphism under allele model (T *vs.* C). The solid diamonds and horizontal lines correspond to the study-specific ORs and 95% CIs. The gray areas reflect the study-specific weight. The hollow diamonds represent the pooled ORs and 95% CIs of the overall population. The vertical solid lines show the OR of 1 and the vertical dashed lines indicate the corresponding pooled OR.

The main results of the current meta-analysis and heterogeneity were summarized in Table 2. In conclusion, we found significant association between *MTHFR* C677T polymorphism and MM susceptibility under allele model (T *vs.* C, OR = 1.17, 95% CI = 1.02–1.34, *P* = 0.03). However, no remarkable association was observed between *MTHFR* C677T polymorphism and MM risk under other four genetic models (TT *vs.* CC, OR = 1.16, 95% CI = 0.98–1.37, *P* = 0.08; TC *vs.* CC, OR = 1.18, 95% CI = 0.96–1.45, *P* = 0.12; TT + TC *vs.* CC, OR = 1.22, 95% CI = 1.00–1.48, *P* = 0.05; TT *vs.* TC + CC, OR = 1.13, 95% CI = 0.98–1.32, *P* = 0.10) (Table 2, Fig. 2).

**Figure 3:**
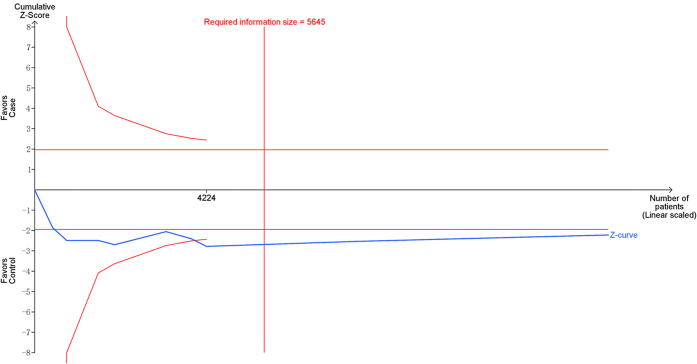
Trial sequential analysis of 9 studies reporting *MTHFR* C677T polymorphism. The required information size was calculated using α = 0.05 (two sided), β = 0.20 (power 80%), D^2^ = 67.0%, a relative risk increase of 16.81% and an event proportion of 38.02% in the control arm. The blue cumulative Z-curve was constructed using a random effects model.

Using the trial sequential analysis (TSA), the required information size is 2823 subjects to demonstrate the issue. Until now, the cumulative Z-curve crossed the trial sequential monitoring boundary and the required information size has been reached, confirming that *MTHFR* C677T polymorphism is associated with increased risk of MM and further relevant trials are unnecessary (Fig. 3). The trial sequential analysis adjusted 95% confidence interval was 0.99 to 1.38.

### Mendelian randomization analysis for the association of *MTHFR* C677T polymorphism with multiple myeloma risk

**Figure 4:**
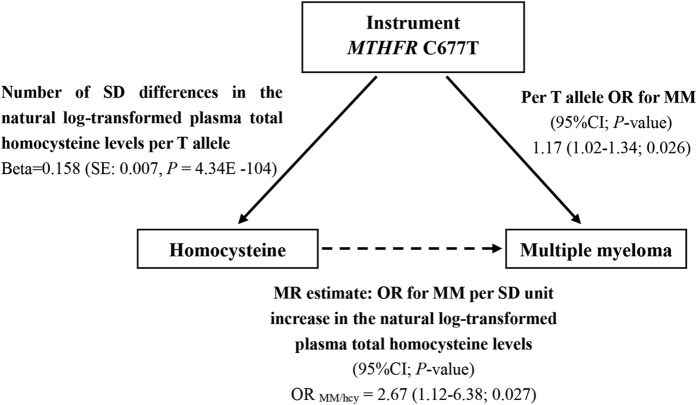
Schematic representation of the Mendelian randomization design. The risk estimate for the association between *MTHFR* C677T polymorphism and MM risk was obtained from the present meta-analysis. The effect of *MTHFR* C677T polymorphism on the SD change in natural log-transformed plasma Hcy levels was obtained from a recent meta-analysis of genome-wide association studies. SE = standard error, SD = standard deviation.

In order to assess the association between genetically increased plasma Hcy level and risk of multiple myeloma, we performed a MR analysis. By combining two pooled estimates, OR _MM/per T-allele_ from a meta-analysis of 9 case-control studies and beta _hcy/per T-allele_ from a meta-analysis of genome-wide association studies by van Meurs and colleagues, we observed that each 1-SD increase in natural-log-transformed plasma Hcy level was associated with a 2.67-fold increased risk of MM (95% CI: 1.12–6.38; *P* = 2.7 × 10^−2^) (Fig. 4). Using the online sample size and power calculator, we had 96.5% power to detect the causal odds ratio.

### Sensitivity analysis and publication bias

**Figure 5:**
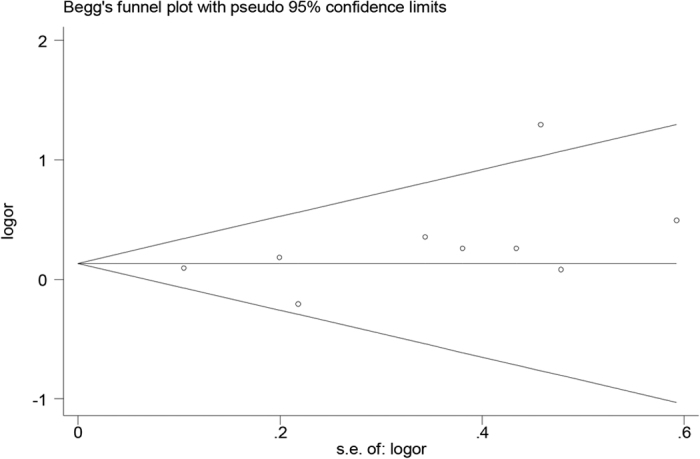
Begg’s funnel plot for the *MTHFR* C677T polymorphism and MM risk (TT *vs.* CT + CC). Each circle represents a separate study for the indicated association. Logor = natural logarithm of OR, s.e. = standard error.

The leave-one-out sensitivity analysis showed that no single study qualitatively altered the pooled ORs, indicating the reliability and stability of our results. Begg’s funnel plot and Egger’s test were performed to evaluate the potential publication bias of literatures. The shape of the funnel plot showed no evidence of obvious asymmetry (Fig. 5). The Egger’s test result did not support the existence of publication bias (TT *vs.* CT + CC, t = 1.53, *P* = 0.169).

## Discussion

Our MR study demonstrated that a genetic increase in natural log-transformed plasma Hcy by 1 SD was associated with a 2.67-fold increased risk of MM, providing strong evidence in support of a causal role of Hcy on MM susceptibility. Since genetic effects on Hcy levels represent differences that generally persist throughout adult life, the estimate of our MR study reflects an effect of Hcy over the course of a lifetime. Meanwhile, our findings are consistent with previous evidence from observational studies that plasma higher Hcy levels influence risk of MM^8^,^9^.To our knowledge, this report is the first to provide evidence for putative causal nature of the association between plasma Hcy and MM.

As we all know, *MTHFR* is a key enzymes of the methylation reaction. *MTHFR* converts 5,10-methyl-enetetrahydrofolate into 5-methyltetrahydrofolate and this reaction provide a methyl for Hcy into methionine in the catalyzed reaction by methionine synthase (MTR) and MTR requires vitamin B12 as a coenzyme^26^. A previous study reported that homozygous mutation of *MTHFR* C677T reduced by approximately 70% of the mean enzyme activity and the heterozygous mutation of *MTHFR* C677T reduced by approximately 35% of the mean *MTHFR* activity^27^. There is no doubt that *MTHFR* C677T polymorphism was related to elevated plasma Hcy levels and lower folate level^28^,^29^,^30^,^31^, which was consistent with recent GWAS meta-analysis^32^.

Hcy, a well-known cardiovascular risk factor, involves in one-carbon methyl group-transfer metabolism^33^. The mechanism has been considered critical for Hcy metabolism in carcinogenesis in terms of DNA synthesis, repair and methylation^34^,^35^. Previous studies have demonstrated that high plasma level of Hcy was associated with risk of a wide range of cancers, such as breast cancer, lung cancer, colorectal cancer and cervical cancer^36^,^37^,^38^,^39^,^40^,^41^. Although there have been no substantial and enough studies to strengthen the view that Hyper-Hcy levels were associated with MM risk, our MR analysis convincingly indicated elevated Hcy was causally associated with increased risk of MM. Therefore, it is necessary to conduct great scale RCTs to assess Hcy-lowering for the treatment and prevention of MM.

Our research has advantages in itself. Firstly, with the application of Mendelian randomization, the effect estimates are closer to the real situation and we are able to overcome potential confounding and reverse causation that may bias evaluations from observational studies. Secondly, Our MR analysis described the association of a lifetime of exposure to Hcy-increasing allele in the general population, whereas observational studies merely provide insights from intervention for shorter periods in individuals at risk. Lastly, the present data from the largest GWAS meta-analysis for Hcy level (44,147 individuals) and from the current meta-analysis for MM risk (2,092 cases and 4,954 controls) have enabled us to more precisely examine our study hypothesis than if we used individual-level data from a small study^32^,^42^.

A few limitations of our study should be considered. First of all, MR estimates which utilize instrumental variables accounting for little variance in a trait tend to be biased towards the null^43^. In this study, we used only one genetic variant as the instrumental variable that influences the plasma Hcy levels. Secondly, it seems difficult for us to exclude the pleiotropy of *MTHFR* C677T polymorphism since data on other clinical parameters across C677T genotypes are rarely provided from most qualified articles, requiring further confirmation. Thirdly, canalization, the process by which compensatory feedback mechanisms attenuate the phenotypic consequences of genetic variation, has been extensively investigated in the circumstance of MR^44^,^45^,^46^. Although compensatory feedback interactions tend to bias results towards the null, the presence of this mechanism would not alter the statistical significance or direction of the effects we found through MR. Finally, considering the differences in minor allele frequencies between populations and other demographic characteristics in the included studies, it is hard to ignore an impact of population stratification.

At last, we have to emphasize this point about the impact of epigenetics on Mendelian randomization as a result of epigenetics gaining recognition as an independent field of study within the last decades. An increasing number of reports suggest that random distribution of epigenetic changes (e.g. gene expression) at conception should be considered on the assumption of MR analysis^47^. Because that, some researchers raised “Two-step epigenetic Mendelian randomization” for establishing the causal role of epigenetic processes in pathways to disease^48^,^49^.

In conclusion, our analysis provides a puissant evidence for a causal role of increased plasma Hcy levels in the etiology of MM. These findings may provide a new insight for further investigating the potential pathogenesis of MM and therapeutic target by decreasing the plasm Hcy levels to prevent the onset and progression of MM. Nevertheless, substantial long-term RCTs assessing the effect of Hcy-lowering on the risk of MM should be carried out in future.

## Methods

### Data on gene association with multiple myeloma risk

To estimate the association of the *MTHFR* C677T polymorphism with multiple myeloma risk, we performed a meta-analysis of case-control studies. We conducted a comprehensive search in Pubmed, Embase, Web of science databases for all eligible studies (updated to Sep 30^th^, 2015) by two authors independently using the following strategies. Key words or terms used for searching were: “methylenetetrahydrofolate reductase” or “*MTHFR*”, “multiple myeloma”, and “polymorphism” or “variation” or “variant” or “mutation” or “genotype” or “allele” or “SNP”, without any restriction on the language. Reference lists of relevant articles were reviewed manually to look for additional studies. For inclusion, studies had to meet the following criteria: (1) evaluation for the association between *MTHFR* C677T polymorphism and multiple myeloma; (2) studies were designed as the case-control type; (3) genotype frequencies for both cases and controls were available. Studies were excluded if: (1) no detailed genotype frequency; and (2) case reports, family-based studies, abstracts, editorials and review articles. When multiple literatures reported the same population, only the most recent one with the largest sample sets was selected for this meta-analysis. Two authors selected the articles independently according to the above criteria. Any uncertainty regarding the eligibility was adjudged by further joint inspection of the publications.

The following data were independently extracted by two investigators from each eligible article according to a fixed protocol: first author’s name, publication year, country and ethnicity of population, genotyping methods, source of control, number of cases and controls, genotype distributions in cases and controls and the HWE in controls (*P* value). If these were not possible, the authors of the publications were contacted via E-mail for more detailed data.

### Data on Gene Association with Hcy

Estimate of the effect sizes of the *MTHFR* C677Tpolymorphism on the plasma Hcy levels was based on the findings of a recent GWAS meta-analysis^32^. The meta-analysis included data from a total of 44,147 white individuals of European ancestry derived from 10 GWAS on Hcy levels.

### Statistical Analysis

#### Meta-analysis

Hardy-Weinberg equilibrium (HWE) of genotypes distribution in the control group was checked by the χ^2^-test and *P* < 0.05 was considered as significant disequilibrium. Studies with controls not in HWE were subjected to a sensitivity analysis. The pooled odds ratios (ORs) with their 95% confidence intervals (95% CIs) were calculated to evaluate the strength of the association between *MTHFR* C677T polymorphism and multiple myeloma risk based on different genetic models: allele model (T *vs.* C), homozygous model (TT *vs.* CC), heterozygous model (CT *vs.* CC), dominant model (TT + CT *vs.* CC), and recessive model (TT *vs.* CT + CC). Statistical heterogeneity between eligible studies was evaluated by using the Cochran’s Q statistic and I^2^ test^50^. *P* < 0.1 and I^2^ exceeding 50% indicated substantial heterogeneity across studies, then a random-effects model was chosen to perform meta-analysis, otherwise, the fixed-effects model was selected. Begg’s funnel plot and Egger’s regression test were used to search for publication bias and a *P* value > 0.05 suggested no significant publication bias have been detected^51^.

#### Trial sequential analysis

Meta-analyses may result in type I errors owing to an increased risk of random error when sparse data are analysed and due to repeated significance testing when a cumulative meta-analysis is updated with new trials^52^,^53^,^54^. We therefore challenged the meta-analyses with the application of trial sequential analysis. Trial sequential analysis is similar to interim analysis in a single trial where the monitoring boundaries are used to decide whether the *P* value is sufficiently small to show the anticipated effect and whether the trial should be terminated early. In the same manner, trial sequential monitoring boundaries can be applied to meta-analyses^16^,^54^,^55^,^56^. TSA depends on the quantification of the required information size. We calculated a diversity-adjusted (D^2^) required information size, since the heterogeneity adjustment with I^2^ underestimates the required information size^57^. TSA was performed with the intention to maintain an overall 5% risk of a type I error and a power of 80%. The required information size was calculated based on a relative risk increase of 16.81% with low risk bias (using the data of allele model). The control event proportion was calculated from the actual meta-analyses.

When the cumulative Z-curve crosses the trial sequential monitoring boundary, a sufficient level of evidence may have been reached and further trials are unnecessary. If the Z-curve does not cross any of the boundaries and the required information size has not been reached, evidence to reach a conclusion is insufficient^58^. We used software Trial Sequential Analysis (version 0.9, http://www.ctu.dk/tsa/) and provided the 95% confidence intervals adjusted for sparse data or repetitive testing, which we describe as the TSA adjusted 95% confidence intervals.

#### Mendelian randomization estimates

We calculated a MR estimate of the effect of the plasma Hcy levels on the risk of multiple myeloma (OR _MM/Hcy_) as log OR _MM/Hcy_ = (log OR _MM/per T-allele_)/beta _Hcy/per T-allele_, as in previous studies^59^,^60^. Log OR _MM/Hcy_ is the (log) increase of multiple myeloma risk by SD unit increase in the natural log-transformed plasma Hcy (MR estimate). Log OR_MM/per T-allele_ is the (log) increase in multiple myeloma risk per allele (gene-multiple myeloma association). Beta _Hcy/per T-allele_ is the number of SD differences in the natural log-transformed plasma Hcy levels per allele (SD/allele) (gene-Hcy association). The standard error of the MR estimate was derived using the Delta method^61^. Using an online sample size and power calculator for Mendelian randomization with a binary outcome (http://spark.rstudio.com/sb452/power/)^62^, we estimate the power, considering sample size, case proportion, odds ratio per SD change in the natural log-transformed plasma Hcy, 0.05 type I error rate, and assuming the variance in Hcy level explained by *MTHFR* C677T polymorphism is R^2^ = 0.01. All *P* values were two sided. All above statistical analyses were performed using STATA software version 12.0 (STATA Corporation, College Station, TX, USA).

## Additional Information

**How to cite this article**: Xuan, Y. *et al.* A Mendelian Randomization Study of Plasma Homocysteine and Multiple Myeloma. *Sci. Rep.* **6**, 25204; doi: 10.1038/srep25204 (2016).

## Figures and Tables

**Table 1:** Main characteristics of studies included in the meta-analysis.

Study	Year	Country	Ethnicity	Genotypingmethods	ControlSource	Sample size(case/control)	Cases			Controls			HWE(P value)
							CC	CT	TT	CC	CT	TT	
Jiang^18^	2014	China	Asia	Microarray	HB	30/157	9	11	10	72	66	19	0.52
Martino^17^	2014	Mixed	Caucasian	TaqMan assay	HB, PB	1264/1797	554	525	185	767	787	243	0.07
Lima^19^	2008	Brazil	Caucasian	PCR-RFLP	PB	123/188	52	57	14	92	79	17	0.99
Moon^20^	2007	Korea	Asia	TaqMan assay	PB	196/434	57	103	36	144	196	94	0.08
Kim^21^	2007	Korea	Asia	PCR-RFLP/Real-time PCR	PB	173/1700	58	80	35	540	863	297	0.13
Chiusolo^22^	2006	Italy	Caucasian	PCR-RFLP	PB	100/100	31	44	25	36	45	19	0.46
Lincz^23^	2003	Australia	Caucasian	PCR-RFLP	PB	90/299	38	44	8	145	133	21	0.20
González-Fraile^24^	2002	Spain	Caucasian	PCR-RFLP	PB	90/79	31	48	11	38	32	9	0.57
González Ordóñez^25^	2000	Spain	Caucasian	PCR-RFLP	Unknown	26/200	5	17	4	92	88	20	0.88

PB, Population-based; HB, Hospital-based; PCR, polymerase chain reaction; RFLP, restriction fragment length polymorphism; HWE, Hardy-Weinberg equilibrium.

**Table 2:** Meta-analysis of the association between *MTHFR* C677T polymorphism and MM risk.

Genetic models	OR (95% CI)	*P* value	Model	I^2^ value (%)
T *vs.* C	1.17 (1.02, 1.34)	0.03	Random	47
TT *vs.* CC	1.16 (0.98, 1.37)	0.08	Fixed	29
TC *vs.* CC	1.18 (0.96, 1.45)	0.12	Random	46
TT + TC *vs.* CC	1.22 (1.00, 1.48)	0.05	Random	47
TT *vs.* TC + CC	1.13 (0.98, 1.32)	0.10	Fixed	20
